# Beyond one-size-fits-all approach: How do various harvesting strategies shape soil microbial diversity in *Larix gmelinii* (Daxinganling larch) forests of the Greater Khingan Mountains?

**DOI:** 10.3389/fmicb.2025.1654005

**Published:** 2025-10-29

**Authors:** Yufeng Wang, Na Ta, Hao Zhang, Min Li, Shengwei Liu, Jiaxing Gong, Yake Song, Rula Sa

**Affiliations:** ^1^College of Forestry, Inner Mongolia Agricultural University, Hohhot, China; ^2^National Field Scientific Observation and Research Station of Greater Khingan Forest Ecosystem, Genhe, China

**Keywords:** harvesting, *Larix gmelinii*, Greater Khingan Mountains/Daxinganling, microbial diversity, functional prediction

## Abstract

This study analyzed the effects of six harvesting strategies, including primary forests (PF), shelterwood cutting (SC), clear cutting (CC), optional cutting with low intensity (OCL), optional cutting with moderate intensity (OCM), and optional cutting with high intensity (OCH), on soil microbial diversity in Daxinganling larch forests of the Greater Khingan Range. The results showed that the dominant bacterial and fungal phyla were similar across the different harvesting strategies. As the intensity of OC increased, the relative abundances of Proteobacteria and Basidiomycota increased, whereas that of Ascomycota decreased. The highest bacterial alpha diversity was observed in the PF sample plots, whereas the highest fungal alpha diversity was observed in the SC sample plots. OCH significantly reduced bacterial alpha diversity (*p* < 0.05), and a negative correlation was observed between OC intensity and bacterial alpha diversity. Harvesting strategies had no significant effect on bacterial or fungal beta diversity. In the harvesting strategy sample plots, 14 biological markers were enriched, including the bacterial family *SC_l_84* and fungal genus *Coniochaeta*. Soil nitrogen, phosphorus, and other physical and chemical properties were significantly correlated with different microbial markers. Soil bacterial and fungal communities have diverse genetic and ecological functions. The bacterial and fungal community networks in the PF sample plots were the most complex and stable. OC reduced the complexity and stability of soil bacterial community networks but had the opposite effect on fungal communities. This study preliminarily analyzed the effects of different harvesting strategies on soil microbial diversity in the Greater Khingan Range *Larix gmelinii forest*, which has practical significance for the functional recovery and protection of the Greater Khingan Range forest ecosystem.

## 1 Introduction

As the largest terrestrial ecosystem on Earth, forests are irreplaceable for maintaining biodiversity, water conservation, and regulating the global carbon balance (Nandal et al., 2023; Hu et al., 2024). Soil microbial communities, dominated by bacteria and fungi, play crucial roles in maintaining forest ecosystems. They profoundly influence ecosystem productivity and stability by driving organic matter decomposition, regulating nutrient cycling, and forming complex symbiotic networks with plant root systems (Delgado-Baquerizo et al., 2020; Li et al., 2025). Harvesting strategies, one of the main forms of anthropogenic disturbance in forests, mainly include SC, OC, CC, and thinning, which aim to obtain timber resources or facilitate forest regeneration (Bont et al., 2022; Repo et al., 2024). However, the impact of harvesting strategies on microbial diversity in forest soils cannot be ignored.

Harvesting activities affect soil microbial community structure and function by directly altering soil physicochemical properties [pH (Melo et al., 2021)], organic matter content (Qiang et al., 2023), temperature, and humidity (Liu et al., 2018a), nutrient substances (Morrison et al., 2016; Liu et al., 2018b), understory microenvironments [light intensity (Zhu et al., 2021)], and plant-microbe interactions (Jog et al., 2014; Cordovez et al., 2021; Wang et al., 2023; Qu et al., 2024).

Different harvesting strategies influence the microbial diversity in forest soils. Reasonable harvesting strategies (moderate thinning and SC) have the potential to maintain or promote soil microbial ecosystems while enhancing forest economic benefits. Thinning contributes to the construction of healthy and stable forest ecosystems by optimizing stand density, wood species composition, and age structure (Zhou et al., 2016). Thinning experiments of different intensities in Chinese fir plantations in the Lishui District, Nanjing Province, China, and oil pine plantations on the Loess Plateau showed that thinning at an appropriate intensity can increase the diversity of plants under the forest and soil microorganisms (Chen et al., 2015; Dang et al., 2018). In a study of secondary forests of *Betula platyphylla Suk* in the Greater Khingan Mountains, SC sample plots had the highest soil bacterial and fungal beta diversity indices, whereas the most intensively damaged fire sample plots had the lowest fungal beta diversity (Zhai et al., 2023). In contrast, CC generally have a negative impact on soil microbial diversity. A meta-analysis showed that CC resulted in 14–33% and 20–40% declines in soil microbial and fungal biomass, respectively (Holden and Treseder, 2013). A study in British Columbia, Canada, showed that soil bacterial and fungal community composition was significantly lower after CC than before, even after 10 or 15 years of plantation-forest restoration. Whole-plant harvesting (with no residues) results in significantly lower soil microbial biomass and diversity than stem harvesting because of the drastically reduced inputs of litter (Hartmann et al., 2009, 2012; Mushinski et al., 2018).

Daxinganling larch (*Larix gmelinii*) is a typical forest species in the cold-temperate region of China and a major community-building species in the forest areas of the Greater Khingan Mountains (Yang et al., 2020; Cheng et al., 2023). It is currently planted in an area of 3 × 10^4^km^2^, accounting for more than 30% of China’s planted forest area (Yang et al., 2010, 2018). However, since the last century, irrational strategies, such as CC and OCH, have proliferated in the region to meet the demand for timber, causing serious damage to forest soil microbial diversity and ecosystem function. In recent years, researchers have conducted preliminary explorations of the impact of harvesting on soil microorganisms in Daxinganling larch. One study showed that CC resulted in a significant decrease in soil microbial carbon and N content, relative abundance of fungi, and Simpson’s diversity index, whereas SC did not (Yang et al., 2020). Another study demonstrated that, compared to PF, Thinning significantly increased the carbon and N content of soil microorganisms in artificial forests, as well as the Shannon and Simpson indices of fungi, but had little effect on bacterial community alpha diversity (*p* > 0.05) (Li et al., 2021). However, the above studies did not systematically analyze the effects of multiple harvesting strategies (SC, OC, and CC) on the diversity of microbial communities in the PF of Daxinganling larch, and research on different OC intensities remains limited.

This study used Daxinganling larch as the research object and preliminarily analyzed the adaptation and response mechanisms of soil microbial diversity to different harvesting strategies. We assumed that (1) different harvesting strategies would affect the soil microbial community, including changes in community composition and diversity. (2) Key biomarkers that respond to different harvesting strategies are related to soil physicochemical properties. Therefore, this study aims (1) to systematically clarify the effects of different harvesting strategies on the alpha and beta diversity of soil bacterial and fungal communities. (2) To explore the effects of different harvesting strategies on the molecular interaction network, genes, and ecological functions of soil microorganisms. (3) To analyze the correlation between the physical and chemical properties of the soil and key biomarkers in sample plots with different harvesting strategies.

## 2 Materials and methods

### 2.1 Location of the study areas

The study was conducted in Inner Mongolia (50° 20’–52° 30’ N, 120° 12’–122° 55’ E), specifically within the Chaocha Forestry. The topography is predominantly characterized by slopes, followed by gentle slopes, with a mean elevation of approximately 950 m (maximum elevation 1210 m). The region experiences a cold-temperate humid monsoon climate, with an average annual temperature of −5.3 °C and an average annual precipitation of 500 mm. The dominant soil type is brown coniferous forest soil, which is sandy, gravelly, and acidic. The forest exhibited high species richness, with Daxinganling larch as the dominant species and broad-leaved conifers, such as Daxinganling white birch (*Betula platyphylla Sukaczev*) and Daxinganling Scotch pine (*Pinus sylvestris var. mongolica litv*). The understory vegetation is well-developed, and the main shrubs include marsh labrador tea (*R. tomentosum*), dahurian rhododendron (*R. mucronulatum*), and Mongolian meadowsweet (*S. pubesscens*), which mainly include wintergreen (*Pyrola calliantha*), Chinese pennisetum (*Pennisetum alopecuroides*), and great burnet (*Sanguisorba officinalis L.*).

### 2.2 Study design

In 2005, six harvesting strategies were set up in the primary forest of Daxinganling larch in Chaocha Forestry: PF, SC, OCL, OCM, OCH, and CC. Each harvesting strategy corresponded to an independent 50 × 50 m plot, totaling six sample plots in the study area. All sample plots had the same ground conditions. Eighteen years after the resumption of harvesting (2023), soil samples were collected, and their physical and chemical properties and microbial diversity were analyzed. Litter was collected and weighed. The characteristics of the sample plots are listed in Table 1.

**TABLE 1:** Characteristics of sample plots information for different harvesting strategies.

						2023		
Harvesting strategy	Sample plot name	Altitude (m)	Slope direction	Harvesting intensity	Post-harvest forest management measures	Constriction (i.e., degree of depression)	Average diameter at breast height (cm)	Tree species composition
Primary forestry (no harvesting)	PF	1100	Dongpo district of central Chongqing municipality, formerly in Sichuan	–	Closure for natural regeneration	0.57	18.78	9 *Larix gmelinii*, 1 *Beula platyphylla Sukaczev*
Shelterwood cutting	SC	1058	Southwestern slope	–	Closure for natural regeneration	0.75	8.72	9 *Larix gmelinii*, 1 *Beula platyphylla Sukaczev*
Optional cutting with low intensity	OCL	1195	Dongpo district of central Chongqing municipality, formerly in Sichuan	30%	Closure for natural regeneration	0.72	8.43	8 *Larix gmelinii*, 2 *Beula platyphylla Sukaczev*
Optional cutting with moderate intensity	OCM	1057	Southwestern slope	50%	Closure for natural regeneration	0.56	8.05	7 *Larix gmelinii*, 3 *Beula platyphylla Sukaczev*
Optional cutting with high intensity	OCH	1121	Dongpo district of central Chongqing municipality, formerly in Sichuan	70%	Closure for natural regeneration	0.43	7.49	6 *Larix gmelinii*, 4 *Beula platyphylla Sukaczev*
Clear cutting	CC	1178	Northwest slope	–	Planting Daxinganling larch	0.48	7.05	8 *Larix gmelinii*, 2 *Beula platyphylla Sukaczev*

### 2.3 Soil sample collection

Six soil sampling points were evenly distributed diagonally across each plot. First, three sampling points were randomly selected, and during sampling, withered material and debris on the soil surface were removed. Soil samples were collected at a depth of 0–20 cm using sterile equipment. After removing visible plant roots, stones, and gravel, the soil samples were air-dried under ventilated and light-free conditions. The air-dried samples were ground and mixed in a sterile mortar, passed through a 2 mm aperture nylon sieve, and then transferred to sterile self-sealing bags and numbered for storage. Part of the samples was used to determine total N, total phosphorus, total potassium, soil organic carbon (SOC), and pH of the soil, while the other part of the samples was immediately placed in an ultra-low-temperature refrigerator at -80°C for storage. Finally, high-throughput sequencing was performed. The remaining three sampling points were used to determine the mass of the fallen leaves in the forest. The area of each sample was 1 m × 1 m in size. All litter in the sample plots was harvested and placed in self-sealing bag.

### 2.4 High-throughput sequencing analysis of soil microorganisms

Total DNA was extracted from the soil samples using the cetyltrimethylammonium bromide method. The purity and concentration were assessed using a NanoDrop 2000 Ultra-Micro Spectrophotometer (Thermo Fisher Scientific, Waltham, MA, USA), and qualified samples were stored at -20°C for subsequent use. The V3 highly variable region of the bacterial genomic 16S rRNA was amplified using PCR (Polymerase Chain Reaction) with the primers 515F (5′-GTGCCAGCMGCCGCGGGTAA-3′) and 806R (5′-GGACTACHVGGGTWTCTAAT-3′). The PCR mix was added to 15 μL Phusion High-Fidelity PCR Master Mix, 0.2 μM primer, and 10 ng genomic DNA template. The PCR conditions were as follows: pre-denaturation at 98 °C for 1 min; 30 cycles at 98 °C for 10 s, 50 °C for 30 s, 72 °C for 30 s, and finally, at 72 °C for 5 min. Primers ITS1F (5′-CTTGGTCATTTAGAGGAAGTAA-3′) and ITS2 (5′-GCTGCGTTCTTCATCGATGC-3′) were used for fungal PCR amplification of the ITS1 region of the genome. The reaction conditions were consistent with those used for the bacteria. The PCR amplicons were electrophoresed on a 2% agarose gel at 100–120 V for 20 min, and the target fragments were recovered using the AxyPrep PCR Purification Kit (E.Z.N.A.^®^ Soil DNA Kit). The purified PCR products were quantified with the Quant-iT PicoGreen dsDNA Assay Kit on a Qubit fluorometric quantification system. Subsequently, library construction was performed. PCR products were quantified using the QuantiFluor™-ST Fluorometer (Promega Biotech, Beijing, China), and the samples were adjusted as needed for sequencing. Sequencing was conducted by Shanghai Majorbio Bio-pharm Technology (Shanghai, China), using an Illumina MiSeq platform (San Diego, CA, USA).

Raw sequencing data were analyzed using the QIIME2 (v2021.11) platform, and each sample data was split from the downstream data according to the barcode and primer sequences using FLASH (version 1.2.11) and Cutadapt software. The data were quality-controlled using fastp software (version 0.23.1), and tag data were obtained from the quality-controlled data. Data quality control was performed using Fastp software (version 0.23.1) to obtain high-quality tagged data. Chimeric sequences were removed by comparison with the Silva and Unite species annotation databases, and effective tags were obtained (Edgar et al., 2011). The DADA2 module (Wang et al., 2021) was used to reduce the noise of the effective tags to obtain Amplicon Sequence Variants (ASVs) and characterization tables. Species annotation was performed using QIIME2, with Silva 138.1 for the bacterial species annotation database and Unite v9.0 for the fungal species annotation database. Rapid multiple sequence comparisons were performed using QIIME2 to determine the phylogenetic relationships of all ASV sequences. Finally, the least amount of data in each sample was used as the criterion for homogenization, and homogenized data were obtained.

### 2.5 Physical and chemical properties of soil and litter quality analysis

Kay type nitrogen determination method, acid-soluble molybdenum antimony colorimetry, and flame photometry were used, respectively, to measure N, P, K content of soil. The electrode potential method was used to determine the soil pH and potassium dichromate oxidation-Spectrophotometry was used to determine the total SOC. The collected litter was brought to the laboratory, dried in an oven at 85 °C until a constant mass was achieved, and then weighed. All the above-mentioned indicators were measured using methods established in previous literature (Liu et al., 2021).

### 2.6 Data processing and statistical analysis

Species abundance histograms of relative abundance in Perl were plotted using the SVG function; species abundance clustering heatmap was plotted using the pheatmap function in R language; Venn diagrams and flower diagrams were generated by the VennDiagram function in R language and SVG function in Perl language, respectively; and the chao1, observed_features, Pielou _e, Shannon, and Simpson diversity indices were generated using QIIME2 software. One-way analysis of variance was performed using SPSS software (version 26.0) to detect the differences between diversity indices (*p* < 0.05); Non-metric multidimensional (NMDS) were performed using R software with the ade4 and ggplot2 packages. Based on the selected data set, Adonis test between different groups was calculated using the psych package (Revelle, 2017). Effect size measurements (LEfSe) software was used to calculate and draw the linear discriminant analysis (LDA) value distribution bar chart and the evolution branch chart (Segata et al., 2011). The functional potential of bacterial communities across different samples was predicted using PICRUSt2 (V2.3.0) based on ASV abundance and the Kyoto Encyclopedia of Genes and Genomes (KEGG) database, whereas the ecological functions of fungal communities were predicted using the FunGuild database. Spearman correlation indices were calculated for the target samples, and filtering conditions were set to (1) remove connections with correlation coefficients <0.6, (2) filter out node self-connections, (3) remove connections with node abundance less than 0.005%, and (4) remove connections with *p*-values of correlation coefficients >0.05. Finally, graphviz-2.38.0 software was used to visualize the microbial community network diagram.

The soil physical and chemical properties and litter mass data were processed using Microsoft Excel 2016. Duncan’s multiple range test was used to analyze the significance of differences between groups, with a significance level of *p* < 0.05. The Mantel test and Pearson’s correlation analysis were performed using the dplyr, ggcor, and ggplot2 packages in R.

## 3 Results

### 3.1 Analysis of soil physical and chemical properties in Daxinganling larch under different harvesting strategies

**FIGURE 1:**
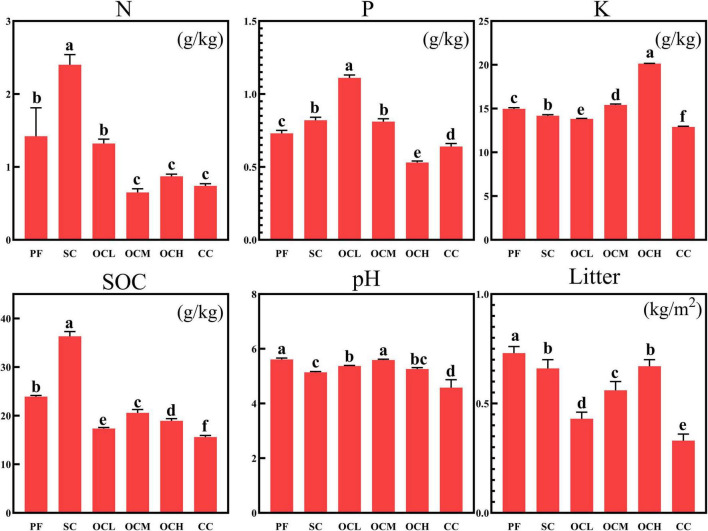
Analysis of soil physical and chemical properties and litter mass in plots with different harvesting strategies. PF, primary forest plots (no harvesting); SC, shelterwood cutting plot; OCL, optional cutting with low intensity plots; OCM, optional cutting with moderate intensity plots; OCH, optional cutting with high intensity plots; CC, clear cutting plots.

Soil physical and chemical properties and litter quality are important indicators for evaluating forest soil health. As shown in Figure 1, the total N and SOC content in SC sample plots were significantly higher than those in other sample plots (*p* < 0.05); the total P content in OCL sample plots was significantly higher than that in other sample plots (*p* < 0.05), and the total P content decreased with increasing OC intensity; the total K content in OCH sample plots was significantly higher than in other sample plots (*p* < 0.05), with total K content increasing with increasing OC intensity; the pH of the PF sample plots was significantly higher than SC, OCL, OCH, and CC sample plots (*p* < 0.05). An analysis of litterfall mass in different harvesting sample plots showed that, as depicted in Figure 1, litterfall mass in PF sample plots was significantly higher than that in other sample plots (*p* < 0.05), and litterfall mass increased with increasing OC intensity. The litterfall mass in the CC sample plots was significantly lower than that in the other sample plots (*p* < 0.05).

### 3.2 Soil microbial ASV counts in Daxinganling larch under different harvesting strategies

**FIGURE 2:**
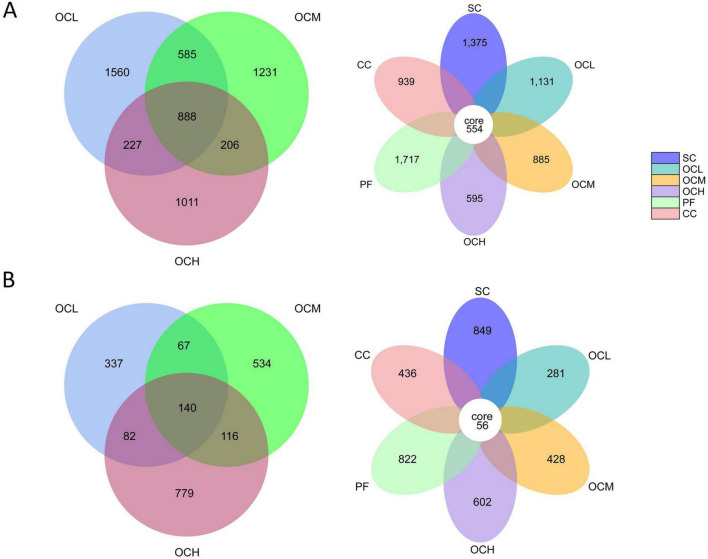
Analysis of soil bacterial and fungal Amplicon Sequence Variants (ASV) counts in sample plots with different harvesting strategies. **(A)** ASV counts of soil bacteria in different harvesting practices. **(B)** ASV counts of soil fungi in different harvesting practices. The CORE numbers in the middle of the petal diagram represent the number of feature sequences common to all sample plots, and the numbers on the petals represent the number of feature sequences specific to the sample plots. The numbers in the overlapping part of the circles in the Wayne diagram represent the number of feature sequences shared among the sample plots, and the numbers without overlapping part represent the number of feature sequences specific to the sample plots.

Soil bacterial and fungal ASV counts were analyzed in the sample plots of the different harvesting strategies (Figure 2). At the bacterial level, the number of ASVs was 1717 in the PF sample plots, 1375 in the SC sample plots, 1131 in the OCL sample plots, 885 in the OCM sample plots, 595 in the OCH sample plots, and 939 in the CC sample plots; 554 ASVs were noted in sample plots with different harvesting strategies; and 888 ASVs were detected in the OCL, OCM and OCH sample plots. At the fungal level, the number of ASVs was 822 in the PF sample plots, 849 in the SC sample plots, 281 in the OCL sample plots, 428 in the OCM sample plots, 602 in the OCH sample plots, and 436 in the CC sample plots. In the sample plots with different harvesting strategies, 56 ASVs were recorded, while the total number of ASVs in the OCL, OCM, and OCH sample plots was 140. Across all sample plots, the number of bacterial ASVs was higher than that of fungal ASVs, indicating a greater bacterial abundance than that of fungi.

### 3.3 Soil microbial relative abundances in Daxinganling larch under different harvesting strategies

Based on the results of species annotation at the bacterial and fungal phylum levels, the top 10 phyla in terms of relative abundance in each plot were selected, and the remaining phyla were grouped as “Others” to create a bar chart of the relative abundance of species.

**FIGURE 3:**
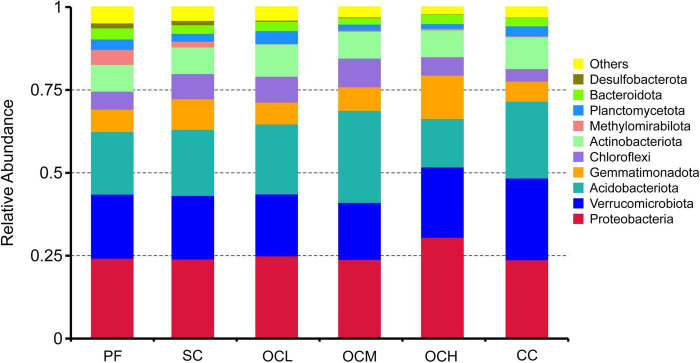
Relative abundance of soil bacteria at the gate level in sample plots with different harvesting practices.

At the phylum level (Figure 3), the dominant phyla in the bacterial communities were similar among the sample plots with different logging methods; however, their relative abundances differed. Proteobacteria, Verrucomicrobiota, and Acidobacteria were highly abundant as co-dominant phyla in all the sample plots. Proteobacteria had the highest relative abundance in all sample plots, Acidobacteria had the highest relative abundance in OCM sample plots, and Verrucomicrobiota had the second highest relative abundance in all sample plots after Proteobacteria and Aidobacteria. Desulfobacterota and Methylomirabilota had lower relative abundances in all sample plots; Desulfobacterota had the highest relative abundance in the PF sample plots and the lowest in CC sample plots, and Methylomirabilota had the highest relative abundance in PF sample plots and the lowest in CC sample plots. The relative abundance of Proteobacteria tended to increase with increasing intensity of OC.

**FIGURE 4:**
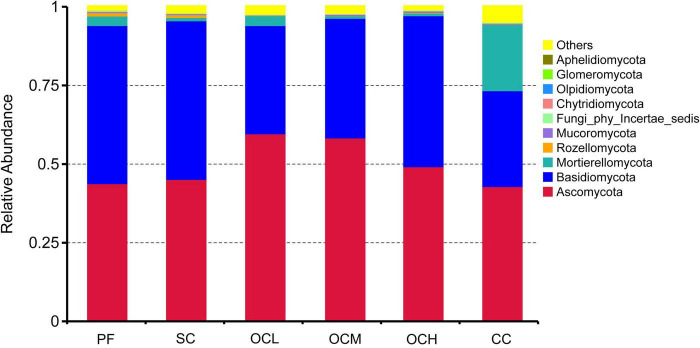
Relative abundance of soil fungi at the phylum level in sample plots with different harvesting practices.

At the fungal phylum level (Figure 4), the dominant phyla of the fungal communities in the different harvesting method sample plots were the same, Ascomycota and Basidiomycota, but their relative abundance varied among the sample plots. The relative abundance of Ascomycota was highest in the CC, OCL, OCM, and OCH sample plots, and that of Basidiomycota was highest in the PF and SC. The sum of the relative abundances of Ascomycota and Basidiomycota was greater than 0.93 in the PF, SC, OCL, OCM, and OCH sample plots, and was 0.73 in the CC sample plots. Mortierellomycota was second only to Ascomycota and Basidiomycota in terms of relative abundance across sample plots, with the highest relative abundance in the CC sample plots and the lowest in the OLM. The relative abundances of fungal phyla, such as Rozellomycota, Mucoromycota, and Chytridiomycota, were low in all sample areas. The relative abundance of Ascomycota decreased with increasing harvesting intensity in the OC, whereas that of Basidiomycota increased with increasing harvesting intensity.

**FIGURE 5:**
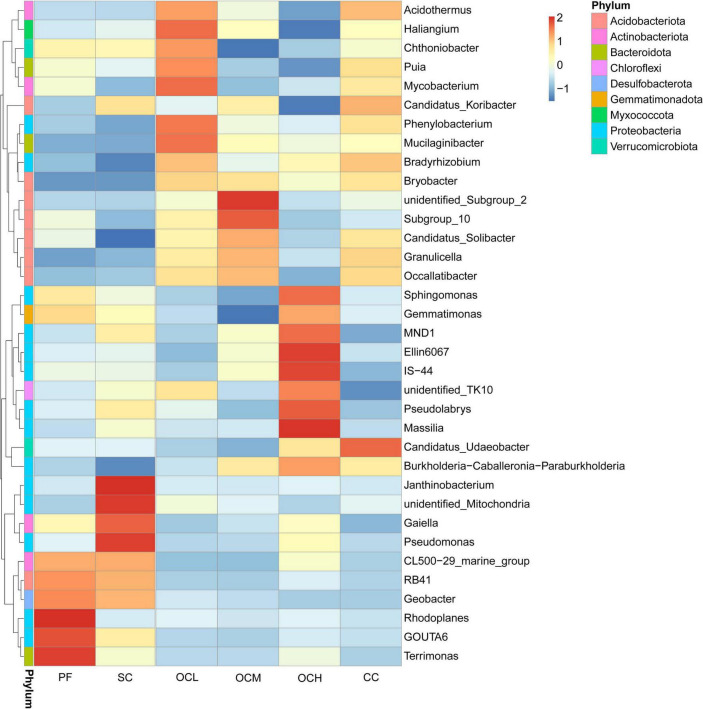
Clustering diagram of species abundance at the bacterial genus level in the sample plots of different harvesting strategies. The horizontal coordinates in the figure are the sample plots names and the vertical coordinates are the species annotation information; the clustering tree on the left side of the figure is the species clustering tree; the values corresponding to the heatmap are the *Z*-values obtained by standardizing the relative abundance of species in each row.

To further investigate the similarities and differences in the composition of bacterial and fungal communities in the sample plots under the different harvesting strategies, the top 35 genera ranked by relative abundance were selected for species abundance clustering analysis based on species annotation and abundance information at the genus level. As shown in Figure 5, the relative abundance of bacteria at the genus level in each sample plot was analyzed using a clustering analysis. The relative abundances of *Rhodoplanes* and *Terrimonas* in the PF sample plots were higher than those in the other sample plots. In the CC sample plots, the relative abundance of *Candidatus_Udaeobacter* was higher than that in the other samples, whereas the relative abundance of *unidentified_TK10* was lower than that in the other samples. In the SC sample plots, the relative abundances of *Janthinobacterium*, *unidentified_Mitochondria*, and *Pseudomonas* were higher than those in the other samples, whereas the relative abundance of *Candidatus_Solibacter* was lower than that in the other samples. The relative abundances of *Haliangium* and *Mycobacterium* were higher than those in the other samples in the OCL sample plots. The relative abundance of unidentified_Subgroup_2 was higher than that in the other samples, whereas the relative abundances of *Gemmatimonas* and *Chthoniobacter* were lower than those in the other samples in the OCM sample plots. The relative abundances of *Massilia*, *Ellin6067*, and *IS-44* were higher than those of the other samples, and the relative abundances of *Haliangium* and *Candidatus_Koribacter* were lower than those of the other samples in the OCH sample plots.

**FIGURE 6:**
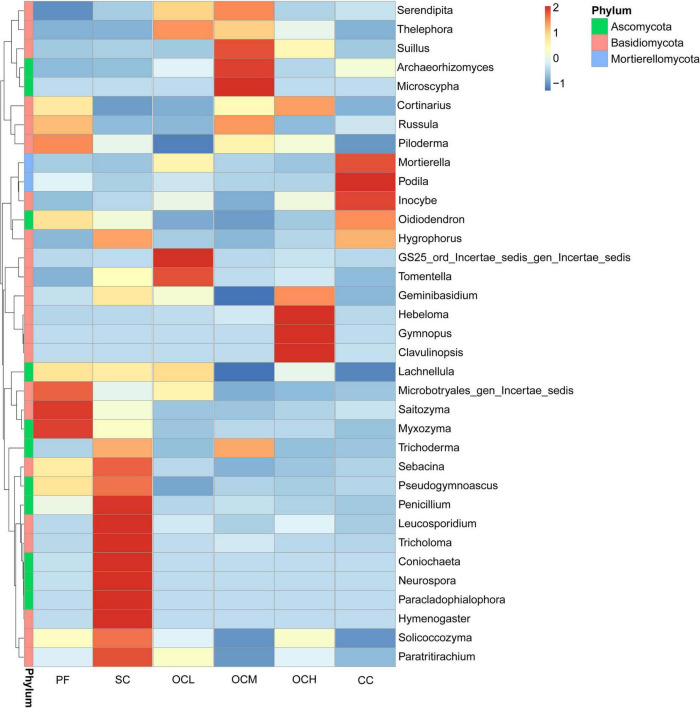
Clustering of species abundance at the fungal genus level in the sample plots of different harvesting strategies.

The relative abundance of fungi at the genus level in each sample plot was analyzed using clustering analysis (Figure 6). The relative abundance of *Microbotryales_gen_Incertae_sedis*, *Saitozyma* and *Myxozyma* in the PF sample plots was higher than that of the other sample plots, while the relative abundance of *Serendipita* was lower than that of the other sample plots. The relative abundances of *Mortierella*, *Podila*, and *Inocybe* were higher in the CC sample plots than in the other samples. In the SC sample plots, the relative abundances of *Penicillium*, *Leucosporidium*, *Tricholoma*, *Coniochaeta*, *Neurospora*, *Paracladophialophora*, *Hymenogaster*, *Sebacina* and *Paratritirachium* were higher than those in the other samples. The relative abundances of *GS_ord_Incertae_sedis_gen_Iencertae_sedis* and *Tomentella* were higher than those in the other OCL sample plots, whereas the relative abundance of *Piloderma* was lower than that of the other samples. The relative abundances of *Suillus*, *Archaeorhizomyces*, and *Archaeorhizomyces* were higher in the OCM sample plots, whereas the relative abundances of *Ceminibasidum* and *Lachnellula* were lower than those in the other samples. The relative abundances of *Hebeloma*, *Gymnopus*, and *Clavulinopsis* were higher than those in OCH sample plots.

### 3.4 Alpha diversity of soil microbial communities in Daxinganling larch under different harvesting strategies

Based on the annotated ASV data, the alpha diversity of the soil bacterial communities in the sample plots of the different harvesting strategies was analyzed (Table 2). The highest index values (chao1, observed_features, Pielou, Shannon, and Simpson) were noted in the PF sample plots, which were higher than those in the other sample plots (*p* < 0.05) and significantly higher than those in the OCH sample plots. The index values showed a decreasing trend with increasing OC intensity. The PF sample plots had the most abundant and uniformly distributed bacterial species and the highest alpha diversity, whereas OCH significantly reduced the bacterial alpha diversity (*p* < 0.05); its alpha diversity was the lowest, and the intensity of OC was negatively correlated with the alpha diversity of the soil bacterial community.

**TABLE 2:** Alpha diversity index of soil bacterial communities in sample plots of different harvesting strategies.

Sample plots name	Chao1	Observed_features	Pielou	Shannon	Simpson
PF	1943.25 ± 69.58a	1892.67 ± 73.21a	0.85 ± 0.01a	9.21 ± 0.11a	1.00 ± 0.00a
CC	1632.82 ± 217.74ab	1606 ± 194.03ab	0.82 ± 0.01b	8.74 ± 0.05ab	0.99 ± 0.00ab
OCL	1606.26 ± 196.71ab	1581.00 ± 170.55ab	0.84 ± 0.00ab	8.90 ± 0.14ab	0.99 ± 0.00a
OCM	1425.13 ± 569.11ab	1397.33 ± 541.63ab	0.83 ± 0.02ab	8.60 ± 0.68ab	0.99 ± 0.00a
OCH	1184.62 ± 74.56b	1176.33 ± 70.81b	0.81 ± 0.01b	8.31 ± 0.02b	0.99 ± 0.00b
SC	1604.38 ± 279.79ab	1572.33 ± 265.51ab	0.82 ± 0.01b	8.69 ± 0.27ab	0.99 ± 0.00ab

Data are expressed as mean ± standard error and different lowercase letters in the same column indicate significant differences (*p* < 0.05) between sample plots of different harvesting strategies, as below.

Based on the annotated ASV data, the alpha diversity of the soil fungal communities in the sample plots under different harvesting strategies was analyzed (Table 3). The SC sample plots had the highest values for all indices (chao1, observed_features, Pielou, Shannon, and Simpson), which were higher than those of the other sample plots. The OCL sample plots had significantly lower values of chao1 and observed_features than the SC sample plots, and the OCM sample plots had the lowest Pielou, Shannon, and Simpson indices. As OC intensity increased, the values of chao1 and observed_features tended to increase, and the values of Pielou, Shannon, and Simpson tended to decrease and then increase again. This indicates that the fungal species in the SC sample plots were the most abundant and evenly distributed, and the alpha diversity was the highest.

**TABLE 3:** Alpha diversity index of soil fungal communities in sample plots of different harvesting strategies.

Sample plots name	Chao1	Observed_features	Pielou	Shannon	Simpson
PF	549.05 ± 62.03a	528.00 ± 58.51ab	0.56 ± 0.04a	5.04 ± 0.43a	0.92 ± 0.03a
SC	566.35 ± 255.33a	547.00 ± 240.81a	0.61 ± 0.10a	5.53 ± 1.29a	0.94 ± 0.05a
OCL	281.85 ± 32.61b	276.67 ± 32.02b	0.59 ± 0.07a	4.75 ± 0.47a	0.93 ± 0.03a
OCM	371.91 ± 151.47ab	348.67 ± 142.92ab	0.52 ± 0.08a	4.00 ± 1.02a	0.88 ± 0.06a
OCH	505.54 ± 121.46ab	483.33 ± 129.83ab	0.56 ± 0.03a	4.94 ± 0.49a	0.90 ± 0.03a
CC	387.53 ± 71.79ab	369.33 ± 59.35ab	0.57 ± 0.03a	4.89 ± 0.34a	0.93 ± 0.02a

Data are expressed as mean ± standard error, and different lowercase letters in the same column indicate significant differences (*p* < 0.05) between sample plots of different harvesting strategies, as below.

### 3.5 Beta diversity analysis of soil microbial communities in Daxinganling larch under different harvesting strategies

**FIGURE 7:**
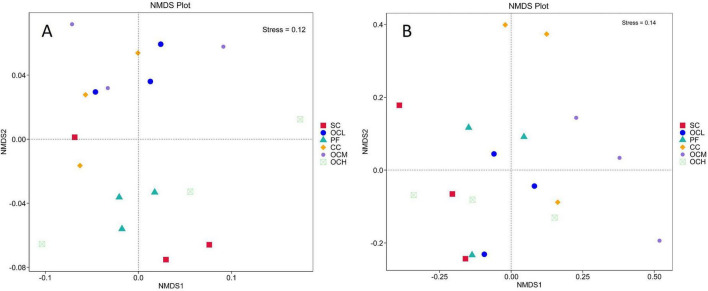
NMDS analysis of bacterial and fungal community structure in sample plots of different harvesting strategies. **(A)** NMDS analysis of bacterial community structure in the sample plots with different harvesting strategies. **(B)** NMDS analysis of fungal community structure in the sample plots of different harvesting strategies. Each point in the graph indicates a sample, the distance between points indicates the degree of difference, and sample plots of the same group are indicated by the same color. When stress is less than 0.2, NMDS can accurately reflect the degree of difference between sample plots.

To study the similarity of soil bacterial and fungal community structures in the sample plots of the different harvesting strategies, NMDS and Adonis analyses were performed. As shown in Figures 7A, B, the stress was less than 0.2, indicating that NMDS could accurately reflect the degree of difference in the bacterial and fungal community structures under different harvesting strategies. As shown in Figure 7A, the different OC intensity, CC, and PF sample plots were far apart, and R^2^ was greater than 0.4 (Table 4), whereas the SC and PF sample plots were closer to each other, and R^2^ was 0.24, indicating that the bacterial community structure of the different intensity of OC and CC sample plots was more different from that of PF sample plots, whereas the SC and PF sample plots were similar in terms of their bacterial community structure. As shown in Table 4, the R^2^ of each harvested sample plot was in the range of 0.3–0.4 compared with the PF sample plots, and the CC and OCM were farther away from the PF sample plots, whereas the other sample plots were closer to the PF sample plots (Figure 7B). This indicates that the fungal community structures of the CC and OCM sample plots differed greatly from those of the PF sample plots, whereas the fungal community structures of the other sample plots were similar to those of the PF sample plots. As shown in Table 4, the Pr values of the differences between the bacterial and fungal groups were greater than 0.05, indicating that the harvesting strategies had no significant effect on the beta diversity of either bacteria or fungi.

**TABLE 4:** Bacterial and fungal community structure of sample plots with different harvesting strategies Adonis intergroup difference analysis.

Comparative combinations	Viruses		Fungi	
	R^2^	Pr (> F)	R^2^	Pr (> F)
SC-OCL	0.32583 (0.67417)	0.1	0.29743 (0.70257)	0.1
SC-PF	0.24909 (0.75091)	0.2	0.3032 (0.6968)	0.1
SC-CC	0.33421 (0.66579)	0.1	0.31259 (0.68741)	0.1
SC-OCM	0.31513 (0.68487)	0.1	0.32635 (0.67365)	0.1
SC-OCH	0.16601 (0.83399)	0.8	0.21748 (0.78252)	0.4
OCL-PF	0.49604 (0.50396)	0.1	0.39673 (0.60327)	0.1
OCL-CC	0.19432 (0.80568)	0.6	0.2606 (0.7394)	0.1
OCL-OCM	0.17221 (0.82779)	0.5	0.29955 (0.70045)	0.1
OCL-OCH	0.31619 (0.68381)	0.1	0.27774 (0.72226)	0.1
PF-CC	0.47984 (0.52016)	0.1	0.31733 (0.68267)	0.1
PF-OCM	0.50687 (0.49313)	0.1	0.33945 (0.66055)	0.1
PF-OCH	0.40141 (0.59859)	0.1	0.36196 (0.63804)	0.1
CC-OCM	0.25413 (0.74587)	0.2	0.25275 (0.74725)	0.2
CC-OCH	0.34726 (0.65274)	0.1	0.3279 (0.6721)	0.1
OCM-OCH	0.29868 (0.70132)	0.1	0.34399 (0.65601)	0.1

R^2^ indicates the degree of explanation of the differences in the sample plots by different subgroups, i.e., the ratio of the subgroup variance to the total variance, the larger the R^2^ indicates that the subgroups have a higher degree of explanation of the differences; Pr indicates the *P*-value, which is less than 0.05, indicating that the credibility of this test is high. Inside the parentheses are the values corresponding to the residual terms.

### 3.6 Analysis of soil bacterial and fungal biomarkers in Daxinganling larch under different harvesting strategies

**FIGURE 8:**
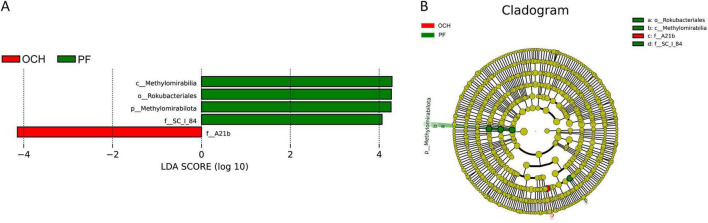
LEfSe analysis of soil bacterial communities in sample plots with different harvesting strategies. **(A)** Histogram of the distribution of bacterial LDA values. **(B)** Branching diagram of bacterial evolution. Species with LDA score greater than a set value (default setting of 4) are shown in the histogram of LDA value distribution.

LEfSe multilevel species difference discriminant analysis was used to identify biomarkers associated with differences in the structure of bacterial and fungal communities across sample plots. At the bacterial level, LEfSe analyses of the bacterial communities in sample plots with different OC intensities (OCL, OCM, and OCH) did not reveal biomarkers with significantly different abundances. LEfSe analysis of bacterial communities in all sample plots (Figure 8) showed that *A21b* was significantly enriched in the OCH sample plots, *Methylomirabilia*, *Rokubacteriales*, *SC_l_84*, and *Methylomirabilota* were significantly enriched in the PF sample plots, whereas the remaining sample plots with other harvesting strategies were not significantly enriched for the biomarkers (Figure 8A). The absolute LDA values of the five biomarkers were all greater than four, indicating highly significant differences (*p* < 0.01), which may be the key biomarkers leading to differences in the bacterial community structure among the PF, CC, and SC, and different intensities of OC sample plots. Cluster analysis of the evolutionary relationships of the above five biomarkers (Figure 8B) showed that the biomarkers in the PF sample plots were categorized into two evolutionary branches (*Methylomirabilota* and *SC_l_84*). *Methylomirabilia* and *Rokubacteriales* were the class and order belonging to *Methylomirabilota*, respectively, whereas *SC_l_84* formed an independent evolutionary branch. *A21b* formed an independent evolutionary branch in the OCH sample plots.

**FIGURE 9:**
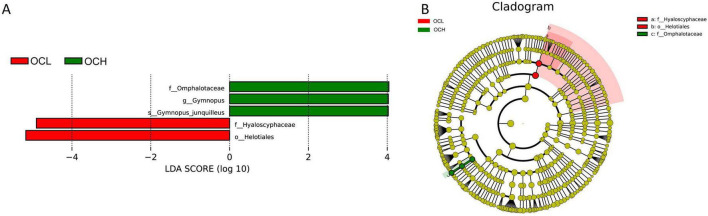
LEfSe analysis of soil fungal communities in sample plots with different selection intensities. **(A)** Histogram of the distribution of fungal LDA values. **(B)** Branching diagram of fungal evolution.

At the fungal level, LEfSe analyses of the fungal communities were performed in sample plots with different OC intensities (OCL, OCM, and OCH) (Figure 9). The results showed that *Hyaloscyphaceae* and *Helotiales* were significantly enriched in the OCL sample plots, *Omphalotaceae*, *Gymnopus*, and *Gymnopus_junquilleus* were significantly enriched in the OCH sample plots, and no biomarkers were significantly enriched in the OCM sample plots (Figure 9A). The absolute LDA values of the five biomarkers were all greater than four, indicating highly significant differences (*p* < 0.01), which may be the key biomarkers leading to differences in the fungal community structure among the OCL, OCM, and OCH sample plots. Cluster analysis of the evolutionary relationships of the five biomarkers showed that two in the OCL sample plots were within the evolutionary branch of *Helotiales* and three in the OCH sample plots were within the evolutionary branch of *Omphalotaceae* (Figure 9B).

**FIGURE 10:**
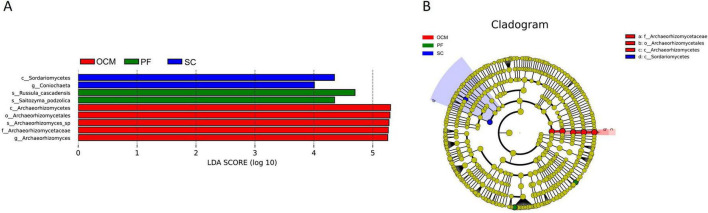
LEfSe analysis of soil fungal communities in sample plots with different harvesting strategies. **(A)** Histogram of the distribution of fungal LDA values. **(B)** Branching diagram of fungal evolution.

LEfSe analysis of the soil fungal communities in the sample plots for each harvesting strategy (Figure 10) showed that *Sordariomycetes* and *Coniochaeta* were significantly enriched in the SC sample plots. *Archaeorhizomycetales*, *Archaeorhizomycetaceae*, *Archaeorhizomyces_sp*, *Archaeor- hizomyces* and *Archaeorhizomycetes* were significantly enriched in OCM sample plots. *Russula_cascadensis* and *Saitozyma_podzolica* were significantly enriched in the PF sample plots, whereas the other harvesting strategy sample plots were not significantly enriched in biomarkers (Figure 10A). The absolute LDA values of the nine biomarkers were all greater than four, indicating highly significant differences (*p* < 0.01), which may be the key biomarkers leading to differences in the fungal community structure among the PF, CC, and OC sample plots. Cluster analysis of the evolutionary relationships of the nine biomarkers showed that five of them in the OCM sample plots were within the evolutionary branch of *Archaeorhizomycetes*, two biomarkers in the SC sample plots were within the evolutionary branch of *Sordariomycetes*, and two biomarkers in the PF sample plots belonged to two separate evolutionary branches (Figure 10B).

### 3.7 Correlation between key microbial markers and physical and chemical properties of the soil

**FIGURE 11:**
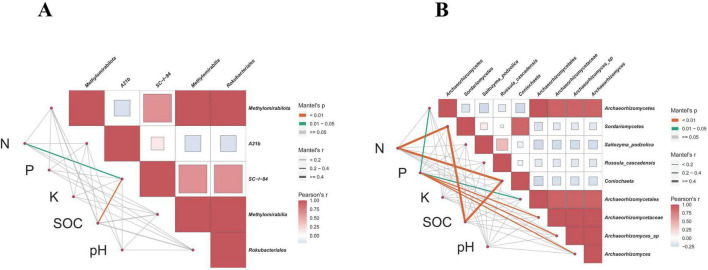
Correlation analysis between key microbial markers in soil and soil physical and chemical properties. **(A)** Bacteria. **(B)** Fungi.

To further clarify the relationship between key microbial markers and soil physicochemical indicators in Xing’an larch forests under different logging methods, the Mantel Test was used for analysis. From a bacterial perspective (Figure 11A), total soil N was significantly correlated with the bacterial family SC_l_84 (*p* < 0.05). SOC was significantly correlated with the bacterial family SC_l_84 (*p* < 0.01). From a fungal perspective (Figure 11B), total soil N was significantly correlated with the fungal class Sordariomycetes and the fungal genus Coniochaeta (*p* < 0.01). Soil total P was significantly correlated with the fungal class Archaeorhizomycetes and the fungal order Archaeorhizomycetales (*p* < 0.05), and was significantly correlated with the fungal family Archaeorhizomycetaceae, fungal species Archaeorhizomyces_sp, and fungal genus Archaeorhizomyces (*p* < 0.01). SOC was highly significantly correlated with the fungal class Archaeorhizomycetes and the fungal genus Coniochaeta (*p* < 0.01).

### 3.8 Predictive analysis of soil microbial functions in Daxinganling larch under different harvesting strategies

**FIGURE 12:**
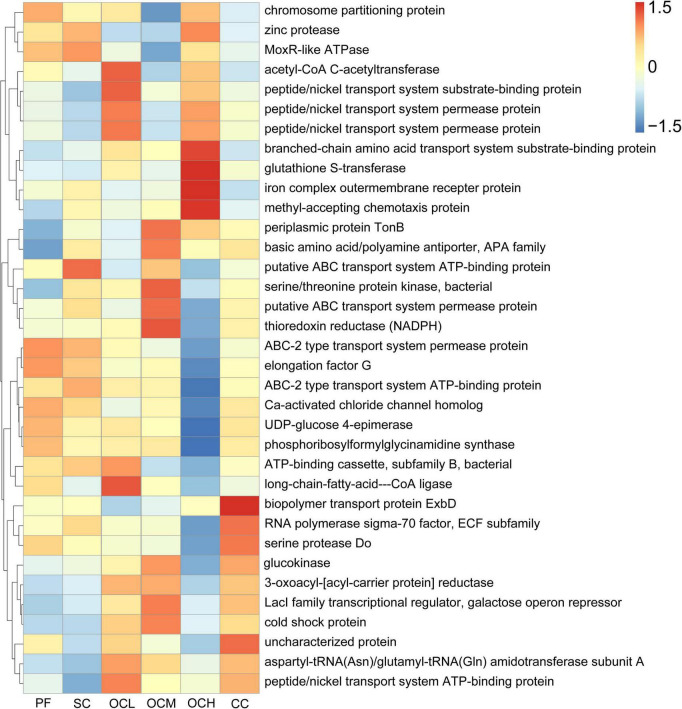
Predictive analysis of soil bacterial functions in sample plots with different harvesting strategies. The horizontal coordinate is the sample plots name, the vertical coordinate is the functional annotation information, and the clustering tree on the left side of the figure is the functional clustering tree; the value corresponding to the heat map is the Z-value obtained by normalizing the relative abundance of each row of function.

Based on the prediction of bacterial gene function using PICRUSt2, the top 35 functions in terms of abundance and abundance information in each sample plots were selected to draw heat maps for comparison with the KEGG database. As shown in Figure 12, the relative abundance of gene functions of ABC-2 type transport system permease protein and elongation factor G was higher in the PF sample plots than in the other sample plots; the relative abundance of gene functions of the putative ABC transport system ATP-binding protein in the SC sample plots were higher than that of other sample plots; the relative abundance of acetyl-CoA C-acetyltransferase, peptide/nickel transport system substrate-binding protein, and long-chain- C-acetyltransferase, peptide/nickel transport system substrate-binding protein, and long-chain- fatty-acid-CoA ligase in the OCL sample plots; in OCM sample plots, the relative abundance of gene function of periplasmic protein TonB, basic amino acid/polyamine antiporter, APA family, serine/threonine protein kinase, bacterial, putative ABC transport system permease protein, and thioredoxin reductase were higher than in the other sample plots; and in the OCH sample plots, the relative abundance of branched- chain amino acid transport system substrate-binding protein, glutathione S-transferase, iron complex outer membrane receptor protein, methyl-accepting chemotaxis protein were higher. The relative abundance of the gene functions of the biopolymer transport protein ExbD and the uncharacterized protein was higher in the CC sample plots than in the other sample plots. Overall, compared to PF, SC, OC, and CC induced the enrichment of different functional bacteria to a certain extent to adapt to the effects of harvesting on soil microbiology in the understory.

**FIGURE 13:**
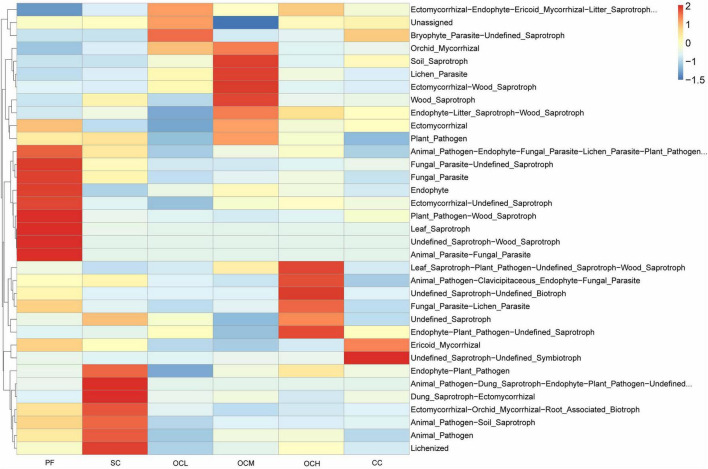
Predictive analysis of soil fungal function in sample plots with different harvesting practices.

Fungal ecological functions were predicted based on FunGuild, and the top 35 functions in terms of abundance and their abundance in each sample plots were selected to draw heat maps. As shown in Figure 13, the fungal ecological functions enriched in the sample plots of different harvesting strategies were different, and there were multifunctional combinations of fungi, such as Animal_Parasite-Fungal_ Paraplot and Leaf_Saprotroph, indicating that the fungi possessed multiple ecological functions in the soil to cope with changes in the soil microenvironment. The relative abundance of eight functions, such as Animal_Parasite-Fungal_Paraplotand Leaf_Saprotroph in the PF sample plots was higher than that in the other sample plots; the relative abundance of seven functions, such as Dung_Saprotroph-Ectomycorrhizal and Lichenized in the SC sample plots was higher than other sample plots; the relative abundance of Bryophyte_Parasite-Undefined_Saprotroph in the OCL sample plots; the relative abundance of four functions, including Soil_Saprotroph and Lichenized_Paraplotin the OCM sample plots; and the relative abundance of the Undefined Saprotroph-Undefined_Saprotroph function and six other functions in the OCH sample plots were higher than other sample plots; the relative abundance of Undefined_Saprotroph-Undefined_Symbiotroph and Ericoid_Mycorrhizal in CC sample plots was higher than other sample plots. Different harvesting strategies induced different ecological functions in the enriched fungal community.

### 3.9 Analysis of soil microbial network interactions in Daxinganling larch under different harvesting strategies

To investigate the covariance characteristics of soil microorganisms, the microbial community covariance network of the sample plots with different harvesting strategies was determined using a one-way covariance network analysis.

**FIGURE 14:**
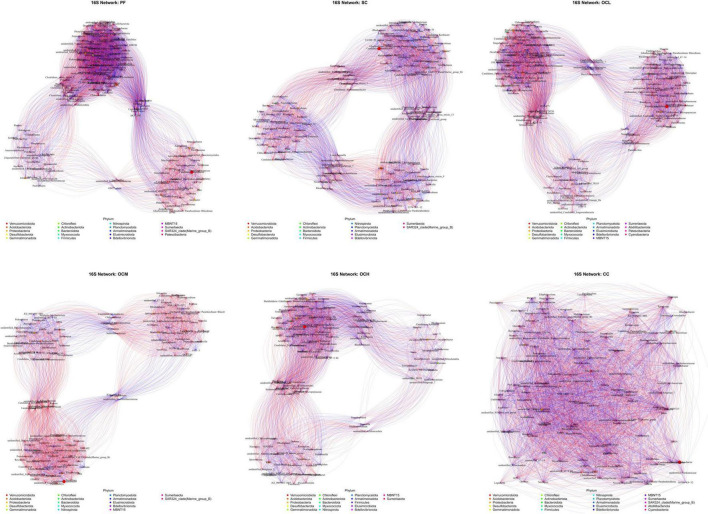
Soil bacterial network analysis of sample plots with different harvesting strategies. Different nodes represent different genera, node size represents the average relative abundance of the genus, nodes of the same phylum are of the same color, the thickness of the connecting lines between the nodes is positively correlated with the absolute value of the correlation coefficient of the species interactions, and the color of the connecting lines corresponds to the positive and negative of the correlation (red is positively correlated, blue is negatively correlated).

Analysis of the bacterial networks in the sample plots subjected to various harvesting strategies (Figure 14 and Table 5) revealed that the PF sample plots exhibited higher node and edge numbers, clustering coefficients, network path densities, and average degrees, along with shorter average path lengths. These findings suggest that the PF bacterial community demonstrated the greatest complexity, largest network size, highest connectivity, and most robust network stability. By analyzing sample plots with different OC intensities, the number of nodes, edges, clustering coefficient, network path density, and average degree of the bacterial network showed a decreasing trend, whereas the average path length showed an increasing trend. This indicates that OC can reduce the complexity and stability of soil bacterial community network. The number of nodes, edges, network path density, and average degree in the CC sample plots were higher than those in the OC sample plots, indicating that the bacterial network in the CC sample plots had more species associations, higher network complexity, and a more stable community.

**TABLE 5:** Topological parameters of soil bacterial and fungal co-occurrence network in sample plots of different harvesting strategies.

Taxon	Sample plots name (of a thing)	Node	Edges	Network diameter	Modularity	Clustering coefficient	Network graph density	Average degree	Average path length
Viruses	PF	153	5583	3	0.31981	0.88683	0.48525	72.98039	1.51475
	SC	141	4510	3	0.36225	0.80565	0.45694	63.97163	1.67001
	OCL	150	4875	3	0.40413	0.87125	0.43624	65	1.61563
	OCM	127	3313	3	0.44082	0.85463	0.41407	52.17323	1.65584
	OCH	122	3621	3	0.31061	0.83161	0.41058	55.36066	1.67067
	CC	139	4654	2	0.32629	0.83126	0.47013	66.96403	1.59193
Fungi	PF	135	4225	3	0.32218	0.85798	0.4975	62.85496	1.62725
	SC	131	4117	3	0.32413	0.84214	0.46711	62.59259	1.66622
	OCL	83	1584	3	0.33472	0.82721	0.46547	38.16867	1.66383
	OCM	100	2452	3	0.30165	0.80301	0.49535	49.04	1.63717
	OCH	128	3953	3	0.39564	0.85555	0.48634	61.76563	1.67913
	CC	100	2235	3	0.38119	0.81485	0.45152	44.7	1.66909

**FIGURE 15:**
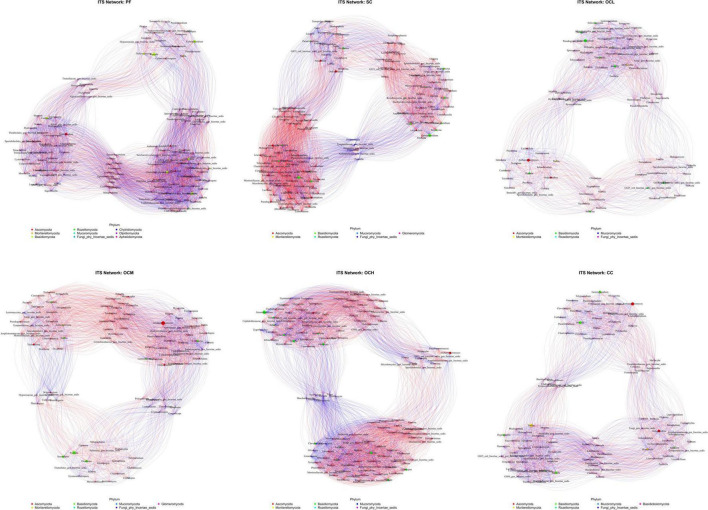
Soil fungal network analysis of sample plots with different harvesting strategies.

Fungal networks in sample plots with different harvesting strategies were analyzed (Figure 15 and Table 5). Within the PF sample plots, the fungal network exhibited a greater number of nodes and edges, along with higher clustering coefficients, network path densities, and average degrees than those in the other sample plots. Conversely, it demonstrated a shorter average path length. These characteristics indicate that the fungal community within the PF sample plots possessed the highest complexity, largest network dimensions, optimal connectivity, and most stable network. By analyzing the sample plots with different OC intensities, the number of nodes, edges, clustering coefficient, network path density, and average degree of the fungal network showed an increasing trend with increasing intensity of OC. This indicates that OC can improve the complexity and stability of the soil fungal community network.

## 4 Discussion

### 4.1 Composition of soil microbial communities in sample plots with different harvesting strategies varies

In this study, six harvesting strategies were used to treat Daxinganling larch, and the soil microbial diversity in the sample plots of each harvesting method was analyzed after an 18-year recovery period. This study showed that in all sample plots, the number of bacterial ASVs was higher than that of fungi (Figure 2). This indicates that bacterial abundance was higher than that of fungi in the inter-root soil of Daxinganling larch, which may be because the root system of Daxinganling larch has a stronger symbiotic interaction with bacteria than with fungi and a higher demand for bacterial populations (Santhanam et al., 2015).

The dominant bacterial phyla in the soil of Daxinganling larch under different harvesting strategies were Proteobacteria, Verrucomicrobiota, and Acidobacteriota. The results of the thinning showed that Proteobacteria, Actinobacteria, and Acidobacteria were the dominant phyla in both the thinning and control sample plots, followed by Verrucomicrobiota (Li et al., 2021), which is consistent with the results of our study. Several studies on soil bacterial diversity in Daxinganling larch have shown that Proteobacteria, Verrucomicrobiota, Acidobacteria, and Verrucomicrobiota are the dominant phyla (Ping et al., 2019; Jia et al., 2020). Our research findings confirmed this result. The relative abundance of Proteobacteria showed an increasing trend with the increase of OC intensity, which might be due to the fact that with the increase of OC, the content of litter left over from felling increases (Figure 1), and the eutrophic phylum Proteobacteria reproduces faster under the high nutrient environmental conditions (Fierer et al., 2007). Ascomycota and Basidiomycota were the dominant fungal phyla in the soils of Daxinganling larches under different harvesting strategies. Ascomycota and Basidiomycota were the dominant fungal phyla in the thinning sample plots of the Daxinganling larch forest (Li et al., 2021), which were the dominant fungal phyla in different forest types of Daxinganling larch (Yang et al., 2017). This indicates that Ascomycota and Basidiomycota, as eutrophic and oligotrophic fungal phyla, respectively, were the primary fungal phyla in Daxinganling larch, and their dominant ecological niches may not have been affected by harvesting strategies.

### 4.2 Diversity of soil microbial communities in Daxinganling larch is closely related to the harvesting strategies

Comparative analysis of the alpha diversity of bacterial communities in the sample plots with different harvesting strategies showed that the values of all alpha diversity indices of bacterial communities in the PF sample plots were significantly higher than those in the OCH sample plots (*p* < 0.05) and higher than those in the other sample plots. This indicates that different harvesting strategies can reduce the number of soil bacterial species. A previous study showed that the Shannon and Simpson values of soil bacterial communities in Daxinganling larch decreased after reasonable thinning, but no significant difference was observed compared with those before the intervention (Li et al., 2021). This suggests that moderate harvesting had little effect on the alpha diversity of soil bacteria in Daxinganling larch, whereas OCH significantly reduced bacterial alpha diversity (*p* < 0.05). In this study, the alpha diversity index values of the CC sample plots were higher than those of the OCH sample plots, which may be due to the large number of Daxinganling larch were planted after harvesting (Table 1), and the number of bacterial species was higher than that of the OCH sample plots after a recovery period of 18 years. A comparative analysis of the alpha diversity of the fungal communities in the different harvested sample plots showed that the alpha diversity indices of the soil fungal communities in the SC sample plots were higher than those in the PF sample plots. This suggests that SC can effectively increase the number of fungal species in the soil. This may be because SC is less damaging to the soil environment of Daxinganling larch than other harvesting strategies, and it can improve the diversity of understory plants by improving understory temperature, humidity, and light (Rottstock et al., 2014), which in turn promotes an increase in the number of soil fungal species and numbers (Qu et al., 2024).

Analysis of the similarity of the bacterial and fungal community structures in the sample plots of different harvesting strategies showed that the bacterial community structures in the SC sample plots did not differ significantly from those in the PF sample plots, consistent with a previous study (Yang et al., 2020). The bacterial community structure in the other sample plots differed from that in the PF sample plots, but the degree of difference decreased with an increase in OC intensity (Table 4), the reasons for which need further investigation. Our study results showed that the fungal community structure of all harvested sample plots differed from that of the PF sample plots, and the degree of difference decreased with an increase in OC intensity, indicating that the response mechanism of the fungal community structure to harvesting was more complex and sensitive. However, the specific reasons for this need to be elucidated in further studies (Zhou et al., 2020).

### 4.3 Biomarkers associated with differences in bacterial and fungal community structure between sample plots with different harvesting strategies

After analyzing the biomarkers related to the differences in the bacterial community structure of different harvesting strategies, it was observed that the bacterial genus *SC_l_84*, which has a denitrification effect, was significantly enriched in the PF sample plots. After long-term natural succession, the humus layer in the PF sample plots is relatively deep, and it is easy to form an anoxic environment suitable for heterotrophic anaerobic bacterial *SC_l_84*, and the continuous large amount of apoplastic input provides a stable carbon source (Holden and Treseder, 2013). However, when PF was harvested, the anaerobic environment of the humus layer was destroyed, the litter was reduced (Hartmann et al., 2009), and the amount of *SC_l_84* decreased significantly. We analyzed the biomarkers associated with differences in fungal community structure in the sample plots under different harvesting practices. The results showed that the five OCM biomarkers belong to *Archaeorhizomycetes*, which are often distributed in plant roots and have saprophytic functions but do not form mycorrhizal structures with roots (Rosling et al., 2011) and may form a mutualistic network with tufted mycorrhizal *Glomerales* to affect plant growth (Choma et al., 2016). The biomarker *Russula_cascadensis*, an ectomycorrhizal fungus at the PF sample plots, can promote plant root growth and nutrient uptake rate (Yang et al., 2025), and the mechanism of its effect on the fungal community structure in the unharvested sample plots requires further exploration.

### 4.4 Effects of different harvesting strategies on soil microbial networks in Daxinganling larch

Microbial co-occurrence network analysis can reveal the interactions between microorganisms, which, in turn, reflect the complexity and stability of the community (De Vries et al., 2018; Ramirez et al., 2018). In our study, the bacterial and fungal community networks were the most complex and stable in the PF sample plots, and the complexity and stability of the bacterial community in the CC sample plots were higher than those in the OC. Several studies on Hainan forests in China have shown that the bacterial and fungal community networks in OC sample plots are more structured than those in PF sample plots, whereas the network structure of CC sample plots is poorer (Chen et al., 2019; Yu et al., 2023). This finding differs from that of our study. First, this is because of the different study areas and forest types. Additionally, all harvested sample plots in this study were artificially planted with a large amount of Daxinganling larch after CC, which recovered the soil bacterial community faster than the OC. In this study, OC reduced the network complexity and stability of soil bacterial communities; however, the opposite was observed for fungal communities. This may be because OCH accelerates the formation of forest windows, promotes apoplastic inputs and nutrient cycling (Figure 1), provides ecological niches for rare fungal taxa, drives the shift from competition to mutualistic symbiosis, and enhances the stability of fungal community networks (Qiang et al., 2023).

## 5 Conclusion

This study provides new insights into the molecular response mechanisms of different harvesting strategies that affect microbial diversity. The highest bacterial alpha diversity was observed in the PF sample plots, whereas the highest fungal alpha diversity was observed in SC sample plots. OCH significantly reduced bacterial alpha diversity (*p* < 0.05), and a negative correlation was observed between OC intensity and bacterial alpha diversity. Harvesting strategies had no significant effect on bacterial or fungal beta diversity. Soil nitrogen, phosphorus, and other physical and chemical properties were significantly correlated with different microbial markers. The bacterial and fungal community networks in PF sample plots exhibited the highest complexity and stability. OC reduced the complexity and stability of soil bacterial networks, while it had the opposite effect on fungal communities, enhancing their network complexity and stability. This study provides a preliminary analysis of how different harvesting strategies influence soil microbial diversity in *L. gmelinii* forests of the Greater Khingan Range, offering practical insights for the functional restoration and conservation of this forest ecosystem.

## Data Availability

The original contributions presented in the study are publicly available. This data can be found here: http://www.ncbi.nlm.nih.gov/bioproject/PRJNA1347439/.
